# Which Classes of Antibiotics Are Associated with the Acquisition of Carbapenemase-Producing Enterobacterales?

**DOI:** 10.3390/life15071072

**Published:** 2025-07-04

**Authors:** Lisa Sadou, Benoît Pilmis, Rasha Eid, Pierre Moenne Locoz, Sophie Lefèvre, Françoise Jauréguy, Vanessa Rathouin, Jean-Ralph Zahar, Laura Foucault-Fruchard

**Affiliations:** 1 Unité de Prévention du Risque Infectieux, Department of Clinical Microbiology, AP-HP, Groupe Hospitalier Paris Seine Saint-Denis, 93000 Bobigny, France; lisa.sadou@aphp.fr (L.S.); pierre.moenne-locoz@aphp.fr (P.M.L.); jeanralph.zahar@aphp.fr (J.-R.Z.); laura.foucault-fruchard@chu-tours.fr (L.F.-F.); 2 Equipe Mobile de Microbiologie Clinique, Hôpitaux Saint-Joseph et Marie-Lannelongue, 75014 Paris, France; 3 Institut Micalis UMR 1319, Université Paris-Saclay, INRAe, AgroParisTech, 91400 Orsay, France; 4 Department of Clinical Microbiology, AP-HP, Groupe Hospitalier Paris Seine Saint-Denis, 93000 Bobigny, France; francoise.jaureguy@aphp.fr; 5 Service de Pharmacie, AP-HP, Groupe Hospitalier Paris Seine Saint-Denis, 93000 Bobigny, France; vanessa.rathouin@aphp.fr; 6 Service Pharmacie, CHU Tours, 37044 Tours, France; 7 Paris Cité Inserm IAME 1137, Université Sorbonne Paris Nord, 75018 Paris, France

**Keywords:** CPE, antimicrobial stewardship, antibiotics, fluoroquinolones

## Abstract

Background: Enterobacterales are among the most frequent causes of healthcare-associated infections and are increasingly affected by antimicrobial resistance. Antibiotic use disrupts the gut microbiota, facilitating colonization by multidrug-resistant organisms, including carbapenemase-producing Enterobacterales (CPE). While animal studies have suggested that certain antibiotic classes may increase the risk of CPE acquisition, clinical data identifying which classes are most implicated remain limited. Methods: We conducted a single-center, retrospective case-control study (2021–2024) comparing antibiotic prescriptions in patients who acquired CPE with those in controls hospitalized in the same unit and during the same risk period but who did not acquire CPE. The objective of this study was to identify which antibiotic classes or pharmacological properties are associated with the acquisition of carbapenemase-producing Enterobacterales (CPE) in hospitalized patients. Results: During the study period, 35 cases and 70 controls were included. Most cases acquired NDM-type metalloenzymes. Before the risk period, 55 patients had received antibiotic therapy. Univariate analysis identified an association between CPE acquisition and the prescription of fluoroquinolones and antibiotics excreted in bile. During the risk period, only metronidazole prescription was significantly associated with CPE acquisition. Our study has several limitations, including the small sample size, the single-center retrospective design, and the lack of molecular typing (e.g., WGS) to confirm potential clonal transmission. Conclusions: In this preliminary study, metronidazole use was associated with an increased risk of CPE acquisition during risk periods. However, these results should be interpreted cautiously and need to be confirmed in larger, multicenter studies. The high exposure of patients to multiple antibiotic classes highlights the importance of strict antibiotic stewardship policies in the current era of global CPE dissemination.

## 1. Introduction

### 1.1. Global Antimicrobial Resistance and Antibiotic Misuse

The acquisition/selection of multiresistant bacteria and intestinal colonization require two steps: first, bacterial transmission and second, antibiotic selection related to antibiotic pressure. Indeed, an important layer of defense is provided by a healthy gut microbiota that protects against colonization of the gut by exogenous microorganisms. This concept is known as ‘colonization resistance’ [1]. Changes in the composition of the gut microbiota can be caused by antibiotic administration, non-antibiotic treatment [2], intestinal infection, or dietary changes [3].

Antibiotics affect the composition and functionality of the human microbiota [4]. The effects of antibiotics on the microbiota can lead to (i) selection of resistant bacteria [5], (ii) dominance of the microbial composition by pathogenic bacteria [6], (iii) loss of bacterial diversity [7], (iv) reduction or even loss of certain bacterial species [8], (v) increased susceptibility to infection, and (vi) risk of new infection and/or recurrence.

### 1.2. Why Focus on Carbapenemase-Producing Enterobacterales (CPE)?

In patients already colonized with multidrug-resistant microorganisms, antibiotic-induced changes in the gut microbiota promote high-density colonization [9]. Epidemiological studies have clearly demonstrated direct relationships between antibiotic consumption and the emergence/dissemination of resistant strains in the hospital [10]. The last decade has seen the emergence and spread of carbapenemases, a plasmid-mediated resistance mechanism, in Gram-negative bacilli, particularly enterobacterales. Although carbapenems are the antibiotic class most frequently cited [11] as being associated with the emergence of carbapenemase-producing Gram-negative bacilli, other antibiotic classes have also been described [12] as being associated with the risk of emergence (i.e., selection/acquisition).

### 1.3. Horizontal Gene Transfer and One Health Perspective

Several previous studies have attempted to identify the antibiotic classes associated with the acquisition of multi-resistant bacteria. Using in vitro models [13], animal models [14], and clinical studies, numerous antibiotic classes have been implicated [15,16], depending on the microbial species and the different resistance mechanisms studied. Several factors also play an important role, such as effects on the anaerobic flora [17], biliary excretion [18], and sub-inhibitory concentrations [19].

### 1.4. Objective of the Study

In fact, the ecological impact of a given class of antibiotics depends on several factors, including the concentration of the drug that reaches the gut microbiota and the susceptibility of the bacterial species. For several decades, all authors have agreed that antibiotics with anti-anaerobic activity play a major role in the resistance phenomenon [20]. More recently, in vivo models have highlighted not only the importance of specific classes, but also the delay between administration and acquisition as a major risk factor [21]. While many authors have highlighted the impact of antibiotics on individual acquisition and infection risk [22], it is surprising to see the low frequency of interventions to control antibiotic prescribing during outbreaks. One possible reason for this [23] could be a lack of understanding of the role of different antibiotic classes in this phenomenon. This study therefore aimed to identify specific antibiotic classes and pharmacokinetic/pharmacodynamic properties—particularly biliary excretion and anti-anaerobic activity [24]—that may be associated with the acquisition of carbapenemase-producing *Enterobacterales* in hospitalized patients.

## 2. Materials and Methods

### 2.1. Study Design and Definitions

We conducted a monocentric retrospective case-control study in the Paris Seine-Saint-Denis hospital group. Between 1 January 2021 and 1 March 2024, all patients hospitalized during risk periods of acquisition defined by the detection of a new case of colonization among contacts of an index patient identified as carrying CPE were included.

Each included case was matched with 2 controls hospitalized in the same unit, during the same period and with the same colonization pressure. All antibiotic prescriptions during hospitalization were collected and collated from medical records.

#### Definitions

Cases were defined as patients who had acquired CPE through contact with an index case. Controls were defined as patients who had been hospitalized in the same unit as the cases for the same period. Cases should have been exposed to an antibiotic prescription and had at least two culture-negative rectal samples.

The “risk” period was defined as the time exposure period during which at least one patient in contact with an index case (i.e., colonized with a CPE isolate) presented the same species and the same resistance mechanism, or only the same resistance mechanism (i.e., enzyme).

Antibiotics were classified according to their biliary excretion and their anti-anaerobic activity according to their spectrum of activity. Antibiotic consumption was measured by the number of antibiotic days.

In addition, antibiotics were considered to be biliary excreted if their biliary elimination was ≥40%. This threshold was based on data from Burdet et al. [24], who estimated the biliary excretion of ceftriaxone to be approximately 40%, compared to 10% for cefotaxime. According to this criterion, the following antibiotics were classified as biliary excreted: metronidazole, rifampicin, macrolides, oxazolidinones, ciprofloxacin, ceftriaxone, meropenem, doxycycline, clindamycin, and trimethoprim-sulfamethoxazole.

The following antibiotics were considered to have anti-anaerobic activity: amoxicillin + clavulanic acid, piperacillin-tazobactam, meropenem, rifampicin, daptomycin, metronidazole, and clindamycin.

Finally, the antibiotics received were also classified and analyzed according to the different classes received.

Antibiotic consumption between cases and controls was compared according to the 2 different periods (before and during the risk period) and included the following: different classes received, total number of antibiotics received, antibiotic duration, number of patients with an antibiotic with biliary elimination, number of patients with an antibiotic with anti-anaerobic activity, and for each of these 2 situations, duration of antibiotic received.

### 2.2. Statistics

Results are presented as median and interquartile range (IQR) or number (%). Variables were compared using the χ^2^ test or Wilcoxon rank-sum. Multivariate analysis of associated variables (*p* ≤ 0.10) in the univariate analysis associated with non-isolation was performed using stepwise inverse logistic regression. Statistical tests were performed using R© software. Differences were considered significant when the *p*-value was 0.05.

### 2.3. Ethics

The study was conducted in accordance with the Declaration of Helsinki and national and institutional standards. The study was approved by the local ethics committee (Comité Local d’Ethique pour la Recherche Clinique des HUPSSD Avicenne-Jean Verdier-René Muret Number CLEA-2024-419).

## 3. Results

During the study period, 15 risk periods were identified in intensive care (n = 6), hepatology (n = 3), geriatrics (n = 2), internal medicine, neurology, cardiology, and infectious diseases, resulting in 35 secondary cases. There were 12 episodes associated with the spread of a metalloenzyme of the NDM type, two episodes associated with a class D carbapenemase of the oxa-48 type and a single episode associated with a class A carbapenemase of the KPC type. This risk period resulted in 1102 contacts. Of these, 300 patients were hospitalized during the same periods as the secondary cases. Two hundred and four contact patients (68%) received an antibiotic prescription during the risk period. Among patients who received antibiotic treatment, only 88 (43%) had undergone at least two rectal examinations for acquisition. 

A total of 105 patients (cases and controls) were included in the study, including 39 ICU patients and 66 non-ICU patients. The mean age of our population was 71 years [59–81], the mean length of hospital stay was 17 days [8–27], and the mean time to collection was 12 days [6.5–20] (Table 1).

### 3.1. Antibiotic Consumption Before the Risk Period

Of the included patients, 55 (52.4%) had received antibiotic therapy during this period and within the previous three months. On average, they had received two [1–2.5] different antibiotic classes, with no differences between cases and controls. When comparing the antibiotic classes prescribed, there was a significant difference in the prescription of fluoroquinolones in patients who had acquired CPE. Also, among the antibiotics received, 31/55 cases (56.3%) had an anti-anaerobic activity and 24/55 cases (43.6%) were bile-excreting. Prior to the risk period, the only statistical difference between cases and controls by univariate analysis was the frequency of consumption of antibiotics with biliary excretion.

### 3.2. Antibiotic Use During the Risk Period

During the risk period, three of the cases who acquired CPE never received antibiotic therapy during their stay. The analysis was therefore performed on the 32 cases and their 63 controls.

In the included population, 15 (15.8%) patients received one antibiotic and 83 (87.3%) received at least two different classes, with a median prescription duration of 17.5 days. Antibiotics with biliary excretion were used in 70 (73.6%) cases, and 73 (76.8%) had anti-anaerobic activity. When comparing colonized and non-colonized patients, there was no significant difference in the mean number of antibiotics received, nor in the mean number of days of biliary or anti-anaerobic antibiotic therapy. There was no difference in the classes of antibiotics prescribed between cases and controls.

## 4. Discussion

### 4.1. Microbiota Disruption and Antibiotic Pressure

Antibiotics administered to patients induce dysbiosis, which contributes to the emergence and acquisition of resistant bacteria. By reducing the bacterial diversity of commensals, they disrupt the resistance to colonization of the microbiome [25]. In our retrospective study, comparing patients who acquired CPE with those who did not, prescription of fluoroquinolone or an antibiotic with biliary excretion before the risk period was associated with acquisition, although this association should be interpreted with caution given the small size and wide confidence intervals.

Despite the small number of patients included, these findings are broadly consistent with existing literature. The role of prior antibiotic exposure—although not controllable in this study—emphasizes the importance of antimicrobial stewardship. This aligns with evidence suggesting that antibiotics can have prolonged effects on the gut microbiota, sometimes lasting up to two years post-exposure. The observed association with biliary-excreted antibiotics before the risk period may reflect the influence of pharmacokinetics on intestinal antibiotic concentrations, a factor known to affect microbiota disruption.

### 4.2. Impact of Metronidazole and Definitions of Anti-Anaerobic Activity

Interestingly, during the risk period, the association was only observed for metronidazole, not for biliary-excreted antibiotics. No significant difference was found in the number of antibiotics received or their duration. This may partly be explained by the difficulty in defining “anti-anaerobic activity” consistently across studies. While some murine and clinical studies suggest that antibiotics active against anaerobes—such as *Bacteroides* spp.—have a strong ecological impact [14], the term itself is not uniformly applied [26].

Similarly, although several studies suggest that biliary-excreted antibiotics contribute to MDRO acquisition [14], our study did not find a consistent association during the risk period. These conflicting results underscore the complexity of assessing the ecological impact of antibiotics and highlight the need for refined classifications—based not only on spectrum but also on pharmacokinetic properties such as biliary excretion and gut-level exposure [24,27].

### 4.3. Interpretation Cautions and Role of Fluoroquinolones

These findings should not be interpreted as evidence that these antibiotics have no ecological effect, but rather as a reflection of the challenges involved in evaluating such effects in a real-world patient population heavily exposed to multiple antibiotics. Effective antimicrobial management thus depends on a better understanding of the microbiota impact of each drug, a task complicated by interindividual variability, underlying disease, and co-medications.

Our data also revealed that among the various antibiotic classes, fluoroquinolones remained strongly associated with CPE acquisition in univariate analysis. Despite the limited sample size, this association reinforces the need to reserve this class for well-justified indications. Fluoroquinolones exert significant ecological pressure due to high intestinal concentrations and have been repeatedly linked to resistance selection [28,29]. In a recent study, both the presence and duration of ciprofloxacin treatment were correlated with an increased number of resistance genes in the microbiota, detectable up to one month after exposure [30].

### 4.4. Antibiotic Use Patterns and the Role of Timing

Finally, we were struck by the frequency and duration of antibiotic use during risk periods: nearly two-thirds of patients received antibiotics, often for extended periods and across multiple classes, regardless of ICU status. This illustrates a clear opportunity for improvement in prescribing practices.

Recent animal models also suggest that the timing of antibiotic administration—especially within the seven days following exposure to a CPE carrier—is critical for acquisition risk [21]. Unfortunately, due to the sample size, we were unable to explore this factor in our study.

### 4.5. Limitations

Our study has several limitations.

Firstly, the small number of patients included and the monocentric nature of the study limit the generalizability of the findings. Larger, multicenter studies are needed to confirm our results.

Secondly, the binary classification of antibiotics into two categories (biliary excretion and anti-anaerobic activity) may have introduced a cognitive bias [31]. Based on prior literature and clinical knowledge, we hypothesized that these two variables were most relevant to acquisition risk, possibly at the expense of other unmeasured or unconsidered factors [32,33].

Third, whole-genome sequencing (WGS) was not performed in either cases or controls, which limits our ability to determine whether the observed events involved clonal transmission or plasmid-mediated dissemination. However, it should be noted that the study was conducted in France, a country with a low prevalence of carbapenemase-producing Enterobacterales (CPE), and no evidence of CPE dissemination in the community to date.

Fourth, while we defined non-acquisition as the presence of two consecutive culture-negative rectal swabs, we acknowledge that this does not fully exclude colonization. The sensitivity of rectal swabbing for the detection of CPE is estimated at 66–78%, depending on methodology, timing, and microbiological techniques [34]. Therefore, some false-negative results are possible despite repeated testing.

Fifth, some odds ratios presented wide confidence intervals (e.g., OR for fluoroquinolone exposure: 90.3 [95% CI: 7.39–3898]), reflecting statistical imprecision related to the small sample size and low event frequency. These findings should be interpreted with caution.

Sixth, although only one variable was statistically significant in univariate analysis, we performed a multivariate model including clinically relevant variables (e.g., ICU length of stay), in response to reviewer suggestion. However, the limited sample size still restricts the power and robustness of this analysis.

## 5. Conclusions

In this single-center study, metronidazole was the only antibiotic significantly associated with CPE acquisition during risk periods. Fluoroquinolones and biliary-excreted antibiotics were also associated with acquisition before the risk period. These findings suggest that specific antibiotic properties may influence colonization risk, but further large-scale studies are needed to confirm these associations and guide stewardship efforts.

## Figures and Tables

**Table 1:** Comparison between patients who acquired CPE and those who did not.

Characteristics	Acquisition (n = 35)	No Acquisition (n = 70)	Univariate Analysis	Multivariate Analysis	
			*p*-Value	*p*-Value	OR (95%IC)
Age (yr) (median, [IQR])	71.3 [66.9–79.9]	69.7 [55.8–79.4]	0.022	0.39	1.02 (0.98–1.06)
ICU admission, n (%)	13 (31.7)	26 (37.1)	>0.99	0.28	0.33 (0.04–2.45)
Hospitalization duration	17 (7.25–30]	15 [8–25]	0.10	0.35	1.03 (0.97–1.10)
**Antibiotic therapy before hospitalization**					
Antibiotic therapy before hospitalization, n (%)	20 (57.1)	35 (50)	0.53	0.68	2.4 (0.71–7.4)
Antibiotic with biliary elimination, n (%)	14/20 (70)	10/35 (28.5)	0.022	0.004	9.38 (1.93–75.2)
Antibiotic with anti-anaerobic activity	11/20 (55)	20/35 (57.1)	0.85	0.26	0.45 (0.10–1.81)
Number of antibiotic family (median, IQR)	2 [1.5–2]	1 [1–3]	0.74	0.95	1.03 (0.40–2.74)
Class of Antibiotics					
BLBLI	6 (17.1)	20 (28.6)	0.19	0.086	0.24 (0.03–1.21)
Aminoglycosides	1 (2.8)	6 (8.6)	0.24	0.54	0.40 (0.01–6.29)
Cephalosporins	11 (31.4)	13 (18.6)	0.15	0.094	4.11 (0.79–23.9)
Fluoroquinolones	8 (22.8)	3 (4.3)	0.005	<0.001	90.3 (7.39–3898)
Anti-staphylococci	2 (5.7)	6 (8.6)	0.59	0.21	0.2 (0.1–10.8)
Metronidazole	2 (5.7)	5 (7.1)	0.78	0.076	0.07 (0.02–1.31)
Carbapenems	4 (11.4)	2 (2.8)	0.086	0.087	30 (0.1–82.2)
Other BL	1 (2.8)	6 (8.5)	0.24	0.13	0.13 (0.2–1.72)
Macrolides	3 (8.6)	4 (5.7)	0.59	0.75	0.68 (0.06–7.25)
**Antibiotic therapy during hospitalization**					
Antibiotic therapy during hospitalization, n (%)	32 (91.4)	63 (90)	0.85	0.72-	0.52 (0.43–3.4)
Antibiotic with biliary elimination, n (%)	23/32 (71.8)	47/63 (74.6)	0.36	0.12	0.34 (0.24–1.77)
Number of antibiotic with biliary elimination (median [IQR])	2 [1–2]	1 [1–2.5]	0.87	0.77	1.22 (0.31–5.17)
Duration of antibiotic with biliary elimination (d) (median [IQR])	7 [2–18]	8 [4.25–14]	0.43	0.44	0.96 (0.84–1.08)
Antibiotic with anti-anaerobic activity	26/32 (81.3)	47/63 (74.6)	0.91	0.72	1.12 (0.87–2.45)
Number of antibiotic with anti-anaerobic activity (median [IQR])	2 [1–3]	2 [1–3]	0.84	0.25	0.45 (0.10–1.78)
Duration of antibiotic with anti-anaerobic activity (d) (median [IQR])	9 [7.25–16.5]	10 [7–20]	0.55	0.91	1.01 (0.89–1.12)
Total antibiotic duration (d) (median [IQR])	7 [3–10.25]	5 [3–8]	0.47	0.57	1.03 (0.92–1.14)
Class of antibiotics					
BLBLI	21 (60)	41 (58.5)	0.89	0.13	1.85 (0.63–5.83)
Aminoglycosides	8 (22.8)	13 (18.6)	0.61	0.75	1.53 (0.28–8.96)
Cephalosporins	20 (57.2)	37 (52.8)	0.68	0.27	0.75 (0.22–2.56)
Fluoroquinolones	7 (20)	17 (24.3)	0.62	0.62	0.53 (0.10–2.35)
Anti-staphylococci	11 (31.4)	25 (35.7)	0.66	0.65	1.34 (0.37–4.76)
Metronidazole	8 (22.8)	8 (11.4)	0.13	0.029	6.94 (1.22–49.1)
Carbapenems	6 (17.1)	21 (30)	0.15	0.12	0.29 (0.05–1.37)
Other BL	4 (11.4)	16 (22.8)	0.15	0.33	0.45 (0.07–2.17)
Macrolides	8 (22.8)	13 (18.5)	0.61	0.78	1.22 (0.28–4.82)

## Data Availability

The original contributions presented in this study are included in the article. Further inquiries can be directed to the corresponding author.

## Abbreviations

The following abbreviations are used in this manuscript:

BLBLI	Beta-lactam/beta-lactam inhibitor
CPE	Carbapenemase producing Enterobacterales
ICU	Intensive care unit
IQR	Interquartile range
MDR	Multidrug resistant
MDRO	Multidrug resistant organism
NDM	New-Delhi metallobetalactamase
